# Peritoneal protein clearance, fluid overload, and cardiovascular events in patients undergoing peritoneal dialysis: a prospective cohort study

**DOI:** 10.1080/0886022X.2025.2461676

**Published:** 2025-02-17

**Authors:** Hongjian Ye, Ruihua Liu, Peiyi Cao, Qunying Guo, Wei Chen, Haiping Mao, Xiao Yang

**Affiliations:** ^a^ Department of Nephrology, The First Affiliated Hospital, Sun Yat-sen University, Guangzhou, China; ^b^ NHC Key Laboratory of Clinical Nephrology (Sun Yat-Sen University) and Guangdong Provincial Key Laboratory of Nephrology, Guangzhou, China

**Keywords:** Peritoneal dialysis, peritoneal protein clearance, fluid overload, ECW/TBW ratio, cardiovascular event

## Abstract

**Background:**

The relationship among higher peritoneal protein clearance (PPCl), fluid overload, and increased risk of cardiovascular (CV) events has not been well clarified in peritoneal dialysis (PD) patients. We aimed to examine their associations in a prospective cohort study.

**Methods:**

Eligible patients were enrolled from a single center, and PPCl was calculated based on the daily dialysate protein loss corrected for serum albumin. Fluid overload was defined as extracellular water (ECW)/total body water (TBW) ≥0.400 measured by bioelectrical impedance analysis (BIA). The primary outcome was CV events.

**Results:**

In total, 351 patients were included in this study. After adjustment for confounders, every 5 mL/day increase in PPCl was independently associated with a 27% higher risk of fluid overload determined by BIA (odds ratio: 1.27, 95% confidence interval (CI): 1.17–1.37). After a median follow-up of 46.8 months, 90 patients (25.6%) experienced CV events. In competing risk models adjusted for confounders, both fluid overload and every 5 mL/day increase in PPCl were independently associated with 70% (subdistribution hazard ratio (SHR):1.70, 95%CI: 1.06–2.74) and 9% (SHR: 1.09, 95%CI: 1.04–1.14) increased risk of CV events, respectively. When fluid overload and PPCl were added simultaneously to the models, PPCl remained a strong independent predictor (SHR: 1.07; 95%CI: 1.03–1.13).

**Conclusions:**

Higher PPCl was independently associated with fluid overload determined by BIA in PD patients. Moreover, higher PPCl was independently associated with an increased risk of CV events.

## Introduction

Peritoneal dialysis (PD) is widely used as an effective treatment for end-stage renal disease (ESRD) in China. PD is considered inferior to hemodialysis in volume control [1,2], due to the large amount of fluid that needs to be instilled into the peritoneal cavity during treatment and the challenging control of peritoneal transport status or lymphatic reabsorption. The high cardiovascular (CV) mortality rates observed in PD patients, in part attributed to the prevalent occurrence of fluid overload, have garnered significant attention [3–6]. The use of bioelectrical impedance analysis (BIA) as a reliable method for volume assessment in dialysis patients has been widely adopted [5, 7–10]. Despite this, our previous randomized controlled trial using BIA-guided fluid management in PD patients revealed no additional advantages in 1-year mortality and CV mortality rates [11]. Therefore, it is necessary to carefully evaluate the potential factors influencing fluid overload in PD patients.

Peritoneal protein clearance (PPCl) reflects the relationship between the peritoneal protein loss (PPL) and serum protein levels in PD patients, and also reflects the three-pore model theory of PD [12,13]. Several studies have demonstrated a close association between PPCl and fluid overload [14–16], indicating that these two conditions may share certain pathophysiological mechanisms, such as comorbidity (e.g., diabetes), higher D/P creatinine, poor residual renal function, and poor nutritional status [2–4, 6, 9, 14–19]. Moreover, higher PPCl has been independently associated with an increased risk of cardiovascular disease (CVD) and mortality in PD patients [13, 20–22].

We were intrigued by the similar pathophysiological basis and adverse effects of PPCl and fluid overload on outcomes in PD patients. Therefore, we conducted a prospective study with a large cohort of PD patients, to investigate the exact relationship between these two factors, and whether their interactions had an impact on CV events. Our study will provide clear results to elucidate the relationship among the two factors. We hypothesize that the interaction between PPCl and fluid overload has a significant worsened impact on patients’ CV outcomes.

## Methods

### Study population

We recruited patients from a single center in our hospital between April and November 2013, who had received PD for at least 3 months and had undergone standardized training at our PD center. All the patients received continuous ambulatory peritoneal dialysis (CAPD) regiment. Commercial dextrose dialysate was used for PD treatment, and patients underwent regular follow-up and adhered to treatment suggestions recommended by their physician. Patients were excluded if they were cycler patients, were unwilling or unable to receive PPCl or BIA examinations, had previously undergone long-term hemodialysis or renal transplantation, were currently on immunosuppressive therapy, had active infection, liver cirrhosis, active hepatitis, malignant tumor, or had experienced an episode of peritonitis within 3 months. The study protocol was approved by the Ethics Committee of the First Affiliated Hospital of Sun Yat-sen University and was conducted in accordance with the ethical principles of the Helsinki Declaration. Written informed consent was obtained from all participants.

### Clinical information and laboratory examinations

We collected baseline demographic and clinical data during clinic visits, including age, gender, body mass index (BMI), comorbid conditions, history of CVD, previous duration on PD, PD dose, and urine volume. Peritoneal equilibration test (PET) results were also reviewed, typically conducted 1–3 months after initiating PD, and were used to classify peritoneal transport types into four groups: low (L), low average (LA), high average (HA), and high (H) [23]. We also assessed biochemistry, high-sensitivity C-reactive protein (Hs-CRP), and adequacy of PD at enrollment, using standard methods such as the weekly urea clearance index (*Kt*/*v* urea) and the weekly total creatinine clearance (CCl) [24]. Total *Kt*/*v* was divided into peritoneal *Kt*/*v* and residual renal *Kt*/*v*, while total CCl was divided into peritoneal CCl and residual renal CCl, considering the contributions of peritoneum and residual renal function to the clearance of small molecules. However, among them, 55 individuals were anuric, and 111 had urinary protein quantification less than 1 g/day. Unlike in chronic kidney disease, urine output in the dialysis population gradually decreases, which means that urinary protein losses will also progressively diminish to none. Urinary protein losses were not included in the analysis due to the fact that a substantial number of patients were anuric at enrollment.

### Definition of fluid overload and evaluation of PPCl

In this study, we utilized the BIA device (InBody720; Biospace, Seoul, South Korea) to assess the fluid volume of our patients when they followed up in the clinic. During the measurement, the patients were instructed to remove their shoes, socks, mobile phone, and metal objects and to place their hands and feet in the sensing position of the device. As in previous studies, we defined fluid overload as an extracellular water (ECW) to total body water (TBW) ratio of ≥0.400, as measured by BIA [5, 11].

To measure PPL, we collected 24-h PD fluid samples from each patient and employed the biuret method. We then calculated the PPCl using the following formula: daily PPL/(serum albumin/0.4783), which has been used in previous studies [13].

### Follow-up and study endpoint

We followed patients until the primary study outcome occurred, or until any reason for withdrawal from PD (such as kidney transplantation, transfer to hemodialysis, or withdrawal from treatment), loss to follow-up, or the end of the study period (31 December 2023). The primary study outcome was defined as the occurrence of the first CVD event, including myocardial infarction, any procedure for arterial revascularization, cardiac sudden death, acute heart failure, hemorrhagic or non-hemorrhagic stroke, hypertensive emergency or subemergency [25–27].

### Statistical analysis

Categorical variables were expressed as frequencies, while continuous variables were presented as mean values with standard deviation (SD) or median values with interquartile ranges (IQRs) after testing for normality. Patients were divided into fluid overload and control groups based on their ECW/TBW ratios, with the former defined as ≥0.400 and the latter <0.400. We used the bivariate correlation test to evaluate the correlation between continuous PPCl and ECW/TBW ratio, and compared the prevalence of fluid overload by the quartiles of PPCl. We employed independent *t*-tests, Chi-square tests, or nonparametric tests, as appropriate, to assess the differences between the two groups. Binary logistic regression or multilinear regression models were utilized to determine whether PPCl was an independent factor of fluid overload or continuous ECW/TBW ratio, respectively, using a forward stepwise method in the multivariate models. Follow-up time was calculated from patient enrollment to primary outcome for time-to-event analysis, with the cumulative hazards for CV events assessed using Kaplan–Meier’s methods and log-rank tests. Competing risk regression was employed for outcome analysis using the Fine and Gray method, with CV events considered principal events while other causes of death and renal transplantation regarded as competing events. In the multivariate models, we adjusted for conventional confounding factors of CVD and factors associated with fluid overload in PD patients, assessed collinearity among all variables, and examined the interaction effect between PPCl and fluid overload on CV event using a competing risk regression model. We also performed time-dependent ROC analysis to observe the continuously time-varying AUCs (area under the curve) of PPCl and fluid overload for predicting CV events at different time points. Statistical analyses were performed using SPSS statistical software (version 23.0, SPSS Inc., Chicago, IL) and R version 3.6.3 (R Project for Statistical Computing, Vienna, Austria), with all *p* values reported as two-tailed and *p* values less than .05 considered statistically significant.

## Results

### Participant characteristics

As shown in Supplemental Figure S1, we screened 601 patients for enrollment. Ultimately, 351 patients met the inclusion criteria and were followed up for a median of 46.8 months. As shown in Table 1 of baseline information, the mean age of the enrolled patients was 47.7 ± 14.3 years old, of which 17.1% were with diabetes. At enrollment, the patients had a median PD treatment duration of 29.2 months, with a mean ECW/TBW value of 0.401 ± 0.014. Based on the study definition, the baseline prevalence of fluid overload in our population was 51.0%.

**Table 1.** Baseline demographic and biochemical data in the overall cohort and patients with or without fluid overload.

Variables	Overall cohort (*n* = 351)	Fluid overload group (*n* = 179)	Control group (*n* = 172)	*p* Value
Male sex (*n*, %)	193 (55%)	86 (48.0%)	107 (62.2%)	.010
Age (years)	47.7 ± 14.3	50.5 ± 15.5	44.8 ± 12.4	<.001
BMI (kg/m^2^)	22.4 ± 3.5	22.5 ± 3.4	22.3 ± 3.3	.516
BSA (m^2^)	1.65 ± 0.17	1.64 ± 0.17	1.65 ± 0.17	.713
History of CVD	43 (12.3%)	26 (14.5%)	17 (9.9%)	.197
Diabetes (*n*, %)	60 (17.1%)	48 (26.8%)	12 (7.0%)	<.001
Primary renal disease	–	–	–	<.001
Glomerular nephritis	235 (67.0%)	113 (63.1%)	122 (70.9%)	–
Diabetic nephropathy	57 (16.2%)	45 (25.1%)	12 (7.0%)	–
Hypertensive nephropathy	32 (9.1%)	13 (7.3%)	19 (11.0%)	–
Others	27 (7.7%)	8 (4.5%)	19 (11.0%)	–
Duration of dialysis at enrollment (months)	29.2 (14.7, 45.3)	33.1 (18.1, 51.4)	25.0 (10.3, 40.9)	<.001
Mean arterial pressure	100 ± 12	101 ± 12	98 ± 11	.023
Dose of PD (L/day)	7.8 ± 1.5	8.1 ± 1.3	7.5 ± 1.6	<.001
Urine volume (mL/day)	350 (70, 800)	150 (10, 500)	600 (300, 1100)	<.001
Ultrafiltration volume (mL/day)	500 (200, 800)	600 (348, 900)	300 (80, 678)	<.001
DPI (g/kg/day)	0.91 ± 0.20	0.88 ± 0.19	0.95 ± 0.20	.001
Total protein (g/L)	68.1 ± 6.5	66.3 ± 6.3	70.0 ± 6.1	<.001
Prealbumin (mg/L)	374 ± 73	387 ± 69	361 ± 74	.001
Albumin (g/L)	38.3 ± 4.0	36.6 ± 3.9	40.1 ± 3.3	<.001
Globulin (g/L)	29.9 ± 4.9	29.7 ± 5.2	30.0 ± 4.6	.501
Peritoneal protein loss (g/day)	4.8 ± 1.6	5.3 ± 1.7	4.2 ± 1.2	<.001
Peritoneal protein clearance (mL/day)	60.9 ± 23.0	70.3 ± 23.9	51.2 ± 17.3	<.001
Hs-CRP (mg/L)	1.43 (0.50, 4.96)	1.61(0.56, 5.95)	1.24 (0.45, 4.06)	.347
Total *Kt*/*v*	2.34 ± 0.66	2.24 ± 0.51	2.47 ± 0.80	.001
Residual renal *Kt*/*v*	0.53 ± 0.77	0.28 ± 0.39	0.80 ± 0.95	<.001
Peritoneal *Kt*/*v*	1.81 ± 0.46	1.94 ± 0.42	1.67 ± 0.46	<.001
Total CCL (L/week/1.73 m^2^)	61.0 (51.7, 79.2)	57.3 (50.0, 69.8)	67.1 (54.3, 90.1)	<.001
Residual renal CCL (L/week/1.73 m^2^)	11.5 (1.5, 34.2)	3.69 (0.19, 18.2)	23.7 (6.9, 52.4)	<.001
Peritoneal CCL (L/week/1.73 m^2^)	46.5 (40.2, 51.8)	48.9 (43.7, 54.1)	43.4 (37.4, 48.8)	<.001
4-h D/P cr	0.68 ± 0.11	0.69 ± 0.10	0.68 ± 0.11	.391
PET type (*n*, %)				.721
Low	9 (2.6%)	5 (3.0%)	4 (2.5%)	–
Low average	113 (32.2%)	58 (34.5%)	55 (34.2%)	–
High average	166 (47.3%)	81 (48.2%)	85 (52.8%)	–
High	41 (11.7%)	24 (14.3%)	17 (10.6%)	–

PET: peritoneal equilibration test; CCL: weekly creatinine clearance; BMI: body mass index; BSA: body surface area; CVD: cardiovascular disease; DPI: daily protein intake.

Values expressed as mean ± SD, median (quartile), or number of patients (%).

### Comparison of the difference between patients with fluid overload and the control group

Compared to the control group, patients with fluid overload had higher proportions of females and diabetes. They were older, had a longer duration of PD, higher ultrafiltration volume, and received a higher dose of PD. Furthermore, they displayed higher PPL and PPCl, and lower residual urine volume. These patients also had poor nutritional status, characterized by lower serum albumin, prealbumin, and daily protein intake (DPI), as well as inferior PD adequacy, characterized by lower total *Kt*/*v* urea and weekly CCl. However, the frequency of history of CVD, Hs-CRP levels, and PET types were not significantly different between the two groups (Table 1).

### Relationship between PPCl and fluid overload and ECW/TBW ratio

In the bivariate correlation analysis, we observed a positive linear relationship between PPCl and ECW/TBW ratio (*r* = 0.432, *p* < .001) (see Supplemental Figure S2). When we stratified patients into four groups based on the quartiles of PPCl, a significant difference in the prevalence of fluid overload was observed. Specifically, only 23.4% of patients in quartile 1 had fluid overload, whereas 79.8% of patients in quartile 4 had fluid overload (*p* < .001) (see Supplemental Figure S3).

We performed multivariate binary logistic regression analysis using a forward stepwise method to identify independent factors associated with fluid overload, while adjusting for age, sex, diabetes, urine volume, DPI, duration of PD at enrollment, dose of PD, residual renal CCl, peritoneal CCl, and D/P creatinine ratio. The results showed that higher PPCl was independently associated with the occurrence of fluid overload (odds ratio = 1.27; 95% confidence interval (CI): 1.17–1.37), along with sex, diabetes, and residual urine volume (Table 2). Considering the collinear relationship between urine volume and ultrafiltration volume for fluid overload, we repeated the analysis, replacing urine volume with ultrafiltration volume in the above models. Surprisingly, we found that PPCl remained associated with fluid overload, independent of the effects of ultrafiltration volume (see Supplemental Table S1).

**Table 2.** Associated factors of fluid overload (ECW/TBW ≥0.400) in the logistic regression models.

Variables	Univariate model		Multivariate model	
	OR (95%CI)	*p* Value	OR (95%CI)	*p* Value
Age (years)	1.03 (1.01–1.05)	<.001	–	–
Sex (M/F)	0.56 (0.37–0.86)	.008	0.32 (0.18–0.56)	<.001
Diabetes (yes vs. no)	4.89 (2.49–9.58)	<.001	4.39 (1.95–9.92)	<.001
Urine volume (every 100 mL/day)	0.87 (0.82–0.91)	<.001	0.87 (0.82–0.93)	<.001
DPI (every 0.1 g/kg/day)	0.82 (0.74–0.92)	.001	–	–
Duration of dialysis at enrollment (months)	1.02 (1.01–1.03)	<.001	–	–
Dose of PD (L/day)	1.31 (1.13–1.53)	.001	–	–
Residual renal CCl (L/week/1.73 m^2^)	0.97 (0.95–0.98)	<.001	–	–
Peritoneal CCl (L/week/1.73 m^2^)	1.07 (1.04–1.09)	<.001	–	–
D/P creatinine ratio (per 0.1 increase)	1.08 (0.88–1.32)	.456	–	–
PPCl (every 5 mL/day)	1.27 (1.19–1.35)	<.001	1.27 (1.17–1.37)	<.001

PPCl: peritoneal protein clearance; DPI: daily protein intake; PD: peritoneal dialysis; CCl: creatinine clearance.

The multivariate binary logistic regression model used a forward stepwise method to explore the independent factors associated with fluid overload, including the following variables: age, sex, diabetes, urine volume, DPI, duration of dialysis at enrollment, dose of PD, residual renal CCl, peritoneal CCl, D/P creatinine ratio, and PPCl.

We further examined the relationship between PPCl and continuous ECW/TBW ratio using a multilinear regression model with a forward stepwise method. We found that PPCl (*β* = 0.341, *p* < .001) and diabetes (*β* = 0.164, *p* < .001) were positively linearly associated with ECW/TBW ratio, while male sex (*β* = −0.187, *p* < .001) and residual urine volume (*β* = −0.256, *p* < .001) were independently and negatively linearly associated with ECW/TBW ratio (Supplemental Table S2).

### PPCl modulates the relationship between fluid overload and CV events

During a median follow-up of 46.8 months (IQR: 24.6, 87.7), 90 patients experienced a first CV event after enrollment. Among these events, 24 (26.7%) were from acute congestive heart failure, 22 (24.4%) were from cardiac sudden death, 17 (18.9%) were from hypertensive emergency or sub-emergency, 16 (17.8%) were from hemorrhagic or non-hemorrhagic stroke, and seven (7.8%) were from myocardial infarction (see Supplemental Figure S1).

**Figure 1.**
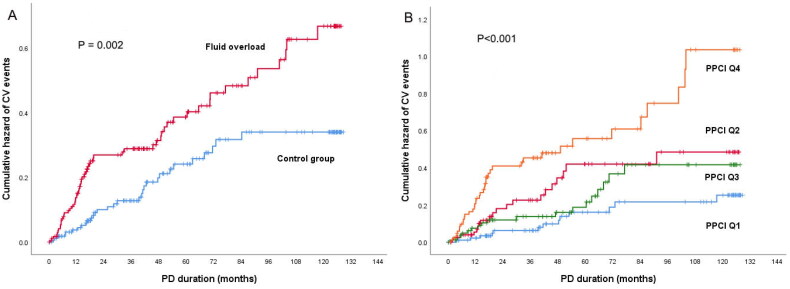
The cumulative risk of CV events in patients with or without fluid overload (A) and patients in different PPCl quartiles (B).

Based on Kaplan–Meier’s survival analysis, patients with fluid overload or higher PPCl had a significantly increased risk for developing CV events (as shown in Figure 1). After adjusting for potential confounders in the competing risk models, higher ECW/TBW ratio (subdistribution hazard ratio (SHR): 1.33; 95%CI: 1.14–1.54) or fluid overload (SHR: 1.70; 95%CI: 1.06–2.74) was independently associated with a higher risk of CV events. Interestingly, when we included PPCl in the above models, the predictive effect of fluid overload for CV events became statistically non-significant, whereas PPCl (SHR: 1.07; 95%CI: 1.03–1.13) remained a strong independent predictor for CV events (Table 3). To assess the interaction-effect between PPCl and fluid overload, we performed an interaction test in the competing risk regression model, and we found a negative result (*p* = .409). There was also no collinearity between the two variables and CV events in the multiple linear regression model (variance inflation factor = 1.23).

**Table 3.** Associations between PPCl and ECW/TBW and outcomes evaluated in the competing risk models.

Variables	Model 1		Model 2		Model 3	
	SHR (95%CI)	*p* Value	SHR (95%CI)	*p* Value	SHR (95%CI)	*p* Value
CV events						
ECW/TBW (every 0.01)	1.36 (1.20–1.54)	<.001	1.33 (1.14–1.54)	<.001	1.23 (1.06–1.45)	.008
Fluid overload (yes vs. no)	2.04 (1.32–3.15)	.001	1.70 (1.06–2.74)	.031	1.31 (0.80–2.16)	.289
PPCl (every 5 mL/day)	1.11 (1.07–1.15)	<.001	1.09 (1.04–1.14)	<.001	1.07 (1.03–1.13)	.002

SHR: subdistribution hazard ratio; CI: confident interval.

Model 1: univariate analysis; model 2: adjusted for age, sex, diabetes, urine volume, respectively; model 3: model 2 + ECW/TBW or fluid overload and PPCl enter together.

### Comparing the time-varying predictive value of PPCl and fluid overload for CV events

**Figure 2.**
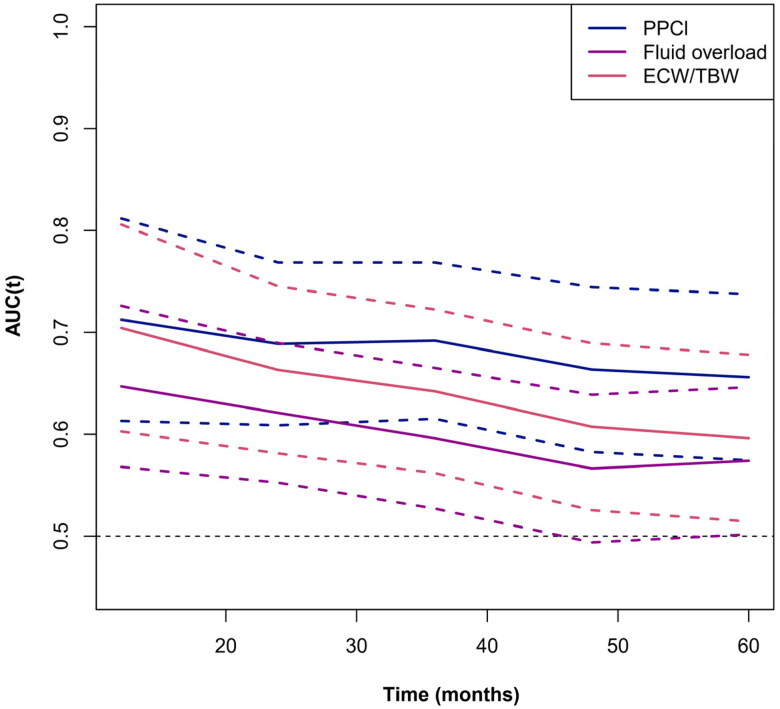
The time-varying AUC values of PPCl and ECW/TBW index for predicting CV events at different time points.

Given the time-varying nature of PPCl and fluid overload in PD patients, we performed a time-dependent ROC analysis to observe the continuously time-varying AUC values of PPCl and fluid overload for predicting CV events at different time points. As shown in Figure 2, all three AUC values decreased to some extent over time. However, the AUC values of PPCl were more stable than those of ECW/TBW ratio or fluid overload.

## Discussion

In this prospective cohort study involving 351 PD patients, we found the prevalence of fluid overload determined by BIA was 51.0%. Baseline PPCl was independently associated with higher risk of fluid overload. Fluid overload was associated with increased risk of CV events, independently of age, sex, diabetes, and residual urine volume. Interestingly, after adjusting for PPCl, the predictive impact of fluid overload on CV events was statistically insignificant, while PPCl remained a robust independent predictor for CV events. PPCl exhibited more stable time-varying AUC values for predicting CV events compared to fluid overload.

Fluid overload is prevalent among PD patients, with a prevalence of 51.0% in our study, while 66.8% in our previous cross-section study [3], and 60% in an European population [4]. There are multiple causes for fluid overload in patients receiving PD. The PD modality itself is believed to be a significant contributor, as the treatment requires fluid to enter the abdominal cavity, leading to a greater fluid burden compared to hemodialysis [1,2]. Similar to previous studies, our data also revealed that diabetes and residual renal function were independently linked to fluid overload parameters [2–4, 17, 28], which suggest that strict blood glucose control and preserving residual renal function may be beneficial in preventing fluid overload in PD patients.

The relationship between fluid overload and clinical outcomes in the PD population is somewhat complex. Previous studies have shown that fluid overload is an independent risk factor for patient survival and CV events in PD [5, 8]. However, an open-labeled, randomized, controlled trial using bioimpedance-guided fluid management in PD reported that bioimpedance guided fluid management can better control volume overload than traditional clinical methods, it failed to improve 1-year patient survival, CV mortality, or technique survival [11]. Though the short follow-up time (1-year survival) may be the main reason for a negative result in the study, there also raises a question: are there any other factors that modify the effect of fluid overload on prognosis? Our study found that PPCl, sex, diabetes, and residual urine volume were independently associated with fluid overload. Previous studies did not consider the independent effect of PPCl on fluid overload when exploring the relationship between fluid overload and outcomes [5, 8]. Moreover, increasing the ultrafiltration volume to address fluid overload often results in an increase in PPCl [29,30], which can attenuate the positive effects of improved fluid overload management. Thus, it is possible that the beneficial impact of addressing fluid overload may be offset by the harmful impact of increased PPCl levels.

The association between PPCl and fluid overload parameter measured by BIA is intriguing. Consistent to previous studies [14–16, 31], our study confirmed the independent association of PPCl with fluid overload. However, these findings were largely based on cross-sectional reports, and the causal relationship remains difficult to establish. It is generally accepted that higher PPCl with a large amount of albumin and other nutrient loss in PD fluid may cause hypoalbuminemia and malnutrition [32–34], which can result in the movement of fluid from intravascular plasma to extravascular interstitial space and a reduction of fluid removal during dialysis, eventually leading to fluid overload. The close association between lower serum albumin and fluid overload has been well-documented [6, 9]. Meanwhile, previous studies have shown that higher PPCl was independently associated with higher peritoneal transport type [13, 18, 29], which is typically characterized by less fluid removal during PD. The reduced fluid removal would prompt physicians to increase more peritoneal exchanges, thereby causing even higher PPCl and exacerbating hypoproteinemia [29]. Moreover, patients with fluid overload often have gastrointestinal edema, which affects protein intake and leads to hypoproteinemia and a worse nutritional status. Our study demonstrated that patients with fluid overload had significantly lower DPI. Therefore, we infer that malnutritional status is likely a key factor in the vicious spiral between PPCl and fluid overload. However, some scholars hold different opinions and emphasize the importance of hydrostatic pressure-induced convection for PPCl, as both the ECW/TBW ratio and ECW excess support the role of an expanded extracellular volume in increasing protein loss in peritoneal dialysate [14,15]. In this theory, the increase in ultrafiltration volume may be the determinant of the increase in PPCl. However, our results showed that the association of PPCl with fluid overload did not depend on the effect of ultrafiltration volume. Interestingly, a longitudinal study found that the initial dialysate to serum total protein correlated with over-hydration (ECW excess), but the association became insignificant thereafter, suggesting that there was no sustained increase in large pore transport with therapy time [16]. Compared with fluid status, the PPCl of PD patients seems to be more stable [16, 29], indicating that the changes in fluid status do not alter such large pore transport status. As demonstrated in our study, PPCl had more stable time-varying AUC values for predicting CV events than fluid overload or the ECW/TBW ratio over time.

Therefore, based on the results in our prospective study, we assume that fluid overload may be an intermediate factor of PPCl affecting the prognosis of PD patients. Of course, we cannot exclude the possibility that individualized peritoneal structure or function determines both PPCl and fluid overload, as a recent study has revealed that AQP1 promoter variant was associated with decreased ultrafiltration and poor outcomes in this population [35].

PPCl reflects the PPL and membrane function, has been proven to be a risk factor for CVD and mortality [5, 13, 20]. Consistent to previous studies, we also proved the independent association between higher PPCl and increased risk of CV events in PD patients. To explain the relevance to CV outcomes, early studies usually considered PPCl as a marker for the severity of systemic vascular disease and local inflammatory injury, as well as the comorbid condition [19, 36–38]. Researchers would also attribute the explanation to the inverse association of PPCl with residual renal function [18, 39]. Generally, patients with poor residual renal function have less urine output, and are more prone to occur fluid overload. They tend to use higher concentrations of dialysate to remove excess water, while increased ultrafiltration volume may be accompanied by more PPL, and resulting in an increase in PPCl. Meanwhile, the close relationship between higher PPCl and fluid overload seems to be overlooked, as our data proved that PPCl was independently associated with higher risk of fluid overload. In addition, PPCl may be also an important nutritional marker for PD patients [18], further exert its negative impact on fluid overload. Heaf et al. observed that patients with larger pore fluid flux had a continuous albumin fall after PD start and consequently caused hypoalbuminemia, which may induce hypercoagulation and endothelial dysfunction in PD patients and contribute to the malnutrition–inflammation–atherosclerosis syndrome [34]. Serum albumin is a strong marker of nutritional status and inflammation, which may also play an important role on the vicious spiral between PPCl and fluid overload, as we discussed above. Our previous study also showed that nutritional status partially dominates peritoneal protein metabolism in PD patients [18]. Higher PPCl might further exert its negative impact on CV prognosis by affecting the peritoneal nutritional metabolism related to fluid overload. Further understanding of the close relationship among PPCl, fluid overload, and CV events may potentially lead to changes in the management of fluid overload in PD. Going forward, it may be more necessary to focus on reducing PPCl in PD patients.

This study has several advantages, including a detailed collection of baseline data from a large sample of PD patients, a prospective follow-up of up to 10 years, and an in-depth observation of the relationship among PPCl, fluid overload, and CV outcomes. However, there are also several limitations. First, we cannot establish a causal relationship between PPCl and fluid overload because they were assessed at the same time when patients were in a relatively stable state of PD. Second, selection bias was inevitable due to the single center nature of the study and the limited scope of the population included. Additionally, we did not intervene in the PPCl or fluid overload, and therefore we still cannot answer the question of which factor is more important in real clinical applications. Moreover, serial measurements of BIA and PPCl were not performed in this study.

In conclusion, higher PPCl was independently associated with fluid overload in PD patients. Higher PPCl was identified as an independent risk factor for long-term CV events in PD patients. Higher PPCl may further negatively impact CV prognosis by affecting fluid overload. Prospective studies should be performed to examine whether reducing PPCl is useful in fluid management and improving CV prognosis in PD patients.

## Supplementary Material

supplement file.docx
