# Supplementary material for: Peritoneal protein clearance, fluid overload, and cardiovascular events in patients undergoing peritoneal dialysis: a prospective cohort study
Source: Ren Fail. 2025 Feb 17;47(1):2461676. doi: 10.1080/0886022X.2025.2461676 (PMC11837914; doi:10.1080/0886022X.2025.2461676)
Supplement: supplement file.docx [file IRNF_A_2461676_SM3564.docx]

**Peritoneal protein clearance, fluid overload, and cardiovascular events in patients undergoing peritoneal dialysis: A prospective cohort study**

**Submit to: Journal of Nephrology**

Hongjian Ye^1,2#^, Ruihua Liu^1,2#^, Peiyi Cao^1,2^, Qunying Guo ^1,2^, Wei Chen^1,2^, Haiping Mao^1,2^, Xiao Yang^1,2*^

^1^ Department of Nephrology, The First Affiliated Hospital, Sun Yat-sen University, 58th, Zhongshan Road II, Guangzhou, 510080, China

^2^ NHC Key Laboratory of Clinical Nephrology (Sun Yat-Sen University) and Guangdong Provincial Key Laboratory of Nephrology, Guangzhou, 510080, China.

^3^ Department of Nephrology, Guangdong Provincial People's Hospital, Guangzhou, 510080, China

^#^ Hongjian Ye and Ruihua Liu contributed equally to this work.

* Correspondent author: Xiao Yang

Department of Nephrology, The First Affiliated Hospital, Sun Yat-sen University, Guangzhou 510080, China.

Tel: 0086-20-87335563. Fax: 0086-20-87769673.

E-mail address: yxiao@mail.sysu.edu.cn

ORCID: 0000-0003-0437-2015

**Table S1. The association between PPCl and fluid overload was independent of ultrafiltration volume and peritoneal CCl**

| **Variables** | **Multivariate model 1** | | **Multivariate model 2** | |
| --- | --- | --- | --- | --- |
|  | **OR (95%CI)** | **P value** | **OR (95%CI)** | **P value** |
| Age (years) | 1.02 (1.00-1.04) | 0.040 | 1.02 (1.00-1.04) | 0.035 |
| Sex (M/F) | 0.43 (0.23-0.80) | 0.008 | 0.33 (0.19-0.58) | <0.001 |
| Diabetes (yes vs. no) | 4.53 (1.79-11.46) | 0.001 | 4.12 (1.70-10.01) | 0.002 |
| Ultrafiltration volume (ml/day) | 1.00 (0.999-1.001) | 0.575 | - | - |
| DPI (every 0.1 g/kg/day) | 0.99 (0.84-1.15) | 0.862 | - | - |
| Duration of dialysis at enrollment (months) | 1.00 (0.99-1.02) | 0.572 | - | - |
| Dose of PD (L/day) | 0.79 (0.58-1.08) | 0.142 | - | - |
| Residual renal CCl( L/week/1.73m^2^ ) | 0.97 (0.95-0.98) | <0.001 | 0.97 (0.95-0.98) | < 0.001 |
| Peritoneal CCl( L/week/1.73m^2^) | 1.02 (0.98-1.07) | 0.291 | - | - |
| D/P creatinine ratio (per 0.1 increase) | 0.39 (0.02-8.34) | 0.546 | - | - |
| PPCl (every 5 ml/day) | 1.25 (1.14-1.36) | <0.001 | 1.24 (1.15-1.34) | < 0.001 |

NOTE. The multivariate binary logistic regression models used a enter (model 1) or forward stepwise (model 2) method to explore whether PPCl was independently associated with fluid overload, including the following variables: age, sex, diabetes, ultrafiltration volume, DPI, duration of dialysis at enrollment, dose of PD, residual renal CCl, peritoneal CCl, D/P creatinine ratio.

Abbreviations: PPCl, peritoneal protein clearance; DPI, daily protein intake; PD, peritoneal dialysis; CCl, creatinine clearance.

**Table S2. Associated factors of continuous ECW/TBW in multilinear regression model**

| **Variables** | **Unstandardized Coefficients** | | **Standardized Coefficients** | **t** | **P value** |
| --- | --- | --- | --- | --- | --- |
|  | **B** | **Standard Error** |  |  |  |
| Sex (male vs. female) | -0.188 | 0.047 | -0.187 | -3.97 | <0.001 |
| Diabetes (yes vs. no) | 0.220 | 0.064 | 0.164 | 3.43 | <0.001 |
| Urine volume (every 100 ml/day) | -0.022 | 0.004 | -0.256 | -5.34 | <0.001 |
| PPCl (every 5 ml/day) | 0.037 | 0.005 | 0.341 | 6.87 | <0.001 |

F=35.41, P<0.001, R^2^=0.304.

NOTE. The multilinear regression model used a forward stepwise method to explore the independent factors associated with fluid overload, adjusting for the following variables: age, sex, diabetes, urine volume, DPI, duration of dialysis at enrollment, dose of PD, residual renal CCl, peritoneal CCl, D/P creatinine ratio, PPCl.

Abbreviations: PPCl, peritoneal protein clearance; DPI, daily protein intake; PD, peritoneal dialysis; CCl, creatinine clearance.


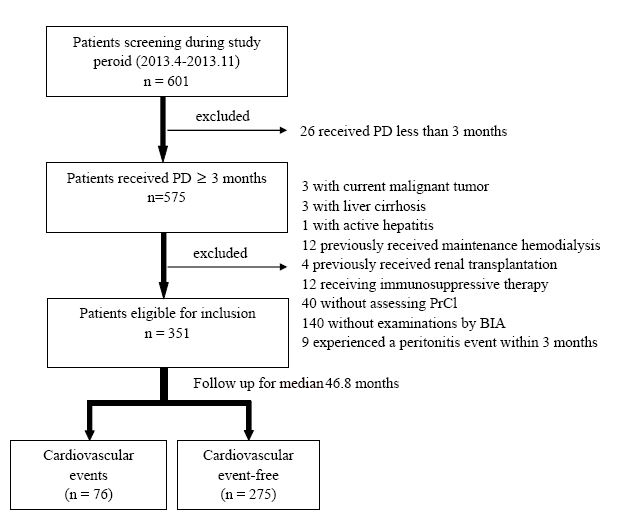


Figure S1. Flow chart of the patient enrollment and follow-up.


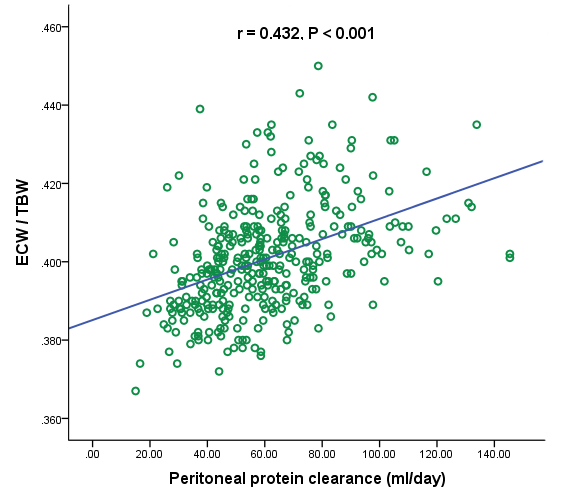


Figure S2. The scatter diagram of the relationship between PPCl and ECW/TBW ratio at baseline (r=0.432, P< 0.001).





Figure S3. The comparison of ECW/TBW index by PPCl quartiles (A) and the prevalence of fluid overload by PPCl quartiles (B)
